# Retraction Note: Platelet-derived growth factor receptor-α and -β promote cancer stem cell phenotypes in sarcomas

**DOI:** 10.1038/s41389-024-00520-7

**Published:** 2024-05-29

**Authors:** Kevin K. Chang, Changhwan Yoon, Brendan C. Yi, William D. Tap, M. Celeste Simon, Sam S. Yoon

**Affiliations:** 1 Department of Surgery, Memorial Sloan Kettering Cancer Center, New York, NY USA; 2 Department of Medicine, Memorial Sloan Kettering Cancer Center, New York, NY USA; 3 Abramson Family Cancer Research Institute, Perelman School of Medicine, University of Pennsylvania, Philadelphia, PA 19104 USA

Retraction to: *Oncogenesis* 10.1038/s41389-018-0059-1, published online 19 June 2018

The authors have retracted this article. After publication, concerns were raised regarding the data presented in the figures. Specifically:

- In Fig. 1A, the SK-LMS-1 CD133 blot image appears to have an inconsistent background
- In Fig. 1C, there appears to be a vertical break in the background of the HT1080 c-Myc blot image, between lanes 1 and 2
- In Fig. 3A, there appears to be a vertical break in the background of the HT1080 Zeb1 blot image, between lanes 1 and 2
- In Fig. 3B, the Migration HT1080 Monolayers image appears highly similar to the Fig. 3D Invasion HT1080 Monolayers and the Invasion SK-LMS-1 imatinib images
- In Fig. 3B, the Migration HT1080 Spheroids image appears highly similar to the Fig. 3D Invasion HT1080 DMSO image and the Fig. 4A MKN-45 Spheroid/DMSO image of [1]
- In Fig. 3B, the Migration SK-LMS-1 Monolayers image appears highly similar to the Fig. 3D Invasion SK-LMS-1 Monolayers image
- In Fig. 3B, the Migration SK-LMS-1 Spheroids image appears highly similar to the Fig. 3D Invasion SK-LMS-1 DMSO image and the Fig. 4A N87 Spheroid/DMSO image of [1]
- In Fig. 3B, the Invasion HT1080 Monolayers image appears highly similar to the Fig. 3D Migration HT1080 Monolayers image
- In Fig. 3B, the Invasion HT1080 Spheroids image appears highly similar to the Fig. 3D Migration HT1080 DMSO image
- In Fig. 3B, the Invasion SK-LMS-1 Monolayers image appears highly similar to the Fig. 3D Migration SK-LMS-1 Monolayers image
- In Fig. 3B, the Invasion SK-LMS-1 Spheroids image appears highly similar to the Fig. 3D Migration SK-LMS-1 DMSO image
- In Fig. 3D, the Invasion HT1080 imatinib image appears highly similar to the Fig. 3G Migration sh.KMT2C of [2]
- In Fig. 3E, there appears to be a vertical break in the background of the DDLS8817 Zeb1 blot image, between lanes 1 and 2
- In Fig. 5B, the CD133/cleaved caspase-3 first panel (from the left) appears highly similar to the third panel of the same row

The authors agree with this retraction.

## References

[1] Yoon C, Park DJ, Schmidt B, Thomas NJ, Lee H-J, Kim TS (2014). CD44 expression denotes a subpopulation of gastric cancer cells in which Hedgehog signaling promotes chemotherapy resistance. Clin Cancer Res.

[2] Cho SJ, Yoon C, Lee JH, Chang KK, Lin J-X, Kim Y-H (2018). KMT2C mutations in diffuse-type gastric adenocarcinoma promote epithelial-to-mesenchymal transition. Clin Cancer Res.

